# Navigating Clinical Efficacy and Legal Boundaries: Implications of Nurse-Led Glycemic Management in Critical Care

**DOI:** 10.3390/healthcare13182313

**Published:** 2025-09-16

**Authors:** Giuseppe Neri, Andrea Bruni, Eugenio Garofalo, Federico Longhini, Vincenzo Bosco

**Affiliations:** Anaesthesia and Intensive Care, Department of Medical and Surgical Sciences, Magna Graecia University, 88100 Catanzaro, Italy; giuseppeneri91@gmail.com (G.N.); andreabruni@unicz.it (A.B.); eugenio.garofalo@unicz.it (E.G.); vincenzo.bosco@unicz.it (V.B.)

**Keywords:** glycemic control, nurse led protocol, insulin, glycemia, intensive care unit

## Abstract

Maintaining optimal blood glucose levels in critically ill patients is a cornerstone of intensive care management. Nurse-led glycemic control protocols, i.e., structured algorithms empowering trained nurses to initiate and adjust insulin therapy, are increasingly adopted to improve the timeliness and consistency of glucose regulation in the Intensive Care Unit. These protocols offer substantial clinical benefits, including faster glucose correction, enhanced adherence to institutional practices, and reduced physician burden. However, their implementation also raises significant legal and ethical concerns. The complexity of critical illness, variability in nursing expertise, and the regulatory boundaries of professional roles may compromise protocol safety and nurse protection if not carefully managed. This paper explores the evidence supporting nurse-led glycemic control, highlighting the risks of both hyperglycemia and hypoglycemia, and examines institutional strategies to mitigate associated challenges. Recommendations include protocol flexibility, rigorous nurse training, structured escalation pathways, legal endorsement, and integration with electronic health records. When grounded in strong clinical governance and legal frameworks, nurse-led protocols can enhance patient outcomes while preserving professional accountability. However, their success depends on a comprehensive, interdisciplinary approach that balances efficiency with individualized care and safeguards all practitioners involved.

## 1. Introduction

Maintaining optimal blood glucose levels is a fundamental aspect of critical care, as both elevated and reduced glucose levels have been associated with higher rates of complications and death among ICU patients. To enhance timely intervention and streamline care processes, numerous intensive care units (ICUs) have implemented nurse-led glycemic control protocols. These protocols authorize nurses to initiate and adjust insulin therapy according to standardized, pre-approved algorithms, reducing the need for continuous physician input on each individual dosage decision [1]. Furthermore, empowering nurses to manage glycemic control aligns with the broader movement toward multidisciplinary, protocol-driven critical care. It frees physician time for more complex decision-making and allows for rapid adjustments during critical windows when delays in insulin titration could lead to adverse events. In resource-limited settings or during periods of physician understaffing (e.g., during pandemics or night shifts), such delegation may be essential to maintaining the standard of care [1].

While nurse-led glycemic control protocols in critically ill patients represent a progressive and evidence-aligned approach to managing glycemic control, their implementation introduces significant clinical and legal implications that must be thoughtfully addressed. After a brief summary of the existing literature, this perspective paper aims to critically reflect on the implications of that evidence for implementation in real-world clinical and legal contexts of nurse-led glycemic control protocols in critically ill patients.

## 2. The Literature Synthesis and Clinical Outcomes

The detrimental effects of abnormal blood glucose levels during critical illness are well established. Elevated glucose levels caused by physiological stress responses have been linked to a higher risk of infections, delayed wound healing, and adverse neurological outcomes [2]. On the other hand, excessive efforts to lower glucose can result in treatment-induced hypoglycemia, a condition independently associated with increased mortality in ICU patients [3,4]. These findings underscore the need for flexible, individualized glycemic control strategies tailored to each patient’s evolving clinical condition.

Nurse-led glycemic control protocols have demonstrated benefits in accelerating glucose correction and promoting uniform adherence to institutional practices. Integration of these protocols into electronic health records or bedside clinical decision-support tools enhances standardization, minimizes the likelihood of manual errors, and reduces inconsistencies in care across different staff and shifts. Evidence from both observational and clinical studies indicates that ICU nurses, when adequately trained and guided by validated insulin infusion algorithms, are capable of achieving and maintaining target blood glucose levels with a high degree of safety and effectiveness [1].

As already highlighted in our previous manuscript [1], a growing body of evidence supports the clinical viability of nurse-led glycemic control protocols in ICUs. Nine studies comparing intervention and control groups found that the proportion of blood glucose values within target range was comparable, 52.0% [36.0; 68.4] in nurse-led protocol groups versus 41.8% [36.2; 69.9] in controls, without a statistically significant difference (*p* = 0.620) [5,6,7,8,9,10,11,12,13]. Across 16 studies [5,6,7,8,10,11,12,13,14,15,16,17,18,19,20], while hyperglycemia only in three [6,14,20], the incidence of hypoglycemia was consistently monitored, while fewer studies addressed hyperglycemia specifically [6,14,20]. Notably, in eight studies reporting average blood glucose levels, patients managed under nurse-led protocols demonstrated lower median glycemia (131 [126; 159] mg/dl) compared to control groups (164 [128; 187] mg/dl), though this difference was not statistically significant (*p* = 0.345) [5,6,8,12,13,15,17,20]. Additionally, two studies assessed glucose variability in detail: Compton et al. found significantly higher nadir glucose levels in the intervention group (93 [79; 105] vs. 80 [66; 95] mg/dl; *p* < 0.001) [5], while Dubois et al. reported more favorable ranges for both lowest and highest glucose levels in the nurse-led cohort, albeit without statistical testing [17].

Importantly, the time required to reach target glycemia, revealing no significant difference between groups (median 10 [7; 13] hours for nurse-led vs. 9 [8; 16] hours for control; *p* = 0.614), suggesting equivalent protocol efficiency [5,6,7,10,11,12,13,15,17,18,19,20,21,22]. Regarding safety and patient-centered outcomes, ICU mortality was not significantly different between groups (RR 0.94, 95% CI 0.80 to 1.11; I^2^ = 25%; *p* = 0.46), a finding reinforced by two studies on long-term mortality at 30 and 90 days (RR 0.91, 95% CI 0.78 to 1.07; I^2^ = 0%; *p* = 0.25) [1]. Finally, ICU length of stay again was found to be similar between nurse-led and control groups (MD −0.24 days, 95% CI −0.66 to 0.17; I^2^ = 23%; *p* = 0.25) [1]. These findings collectively demonstrate that nurse-led glycemic control protocols are not only clinically comparable to physician-led approaches in achieving glycemic targets and minimizing adverse events, but also safe and operationally feasible when implemented within structured, protocol-driven environments.

While the literature generally supports the safety and feasibility of nurse-led glycemic control protocols, there is a significant heterogeneity among the studies in the literature, particularly in terms of protocol design, insulin administration methods, and target glycemic ranges. Moreover, the overall methodological quality of the evidence base remains low since most of the studies are not randomized controlled trial, sometimes lacking control groups, undermining the strength of causal inferences. Among studies, populations are also heterogeneous, often including critically ill patients with and without a prior history of diabetes, raising concerns about the influence of pre-existing metabolic conditions on outcome variability [1]. These limitations, combined with clinical and methodological diversity, necessitate that conclusions be interpreted within the specific contexts and settings of each study, and underscore the need for more rigorous, standardized research in this field.

## 3. Limitations of Nurse-Led Glycemic Control Protocols

Although nurse-led glycemic control protocols offer some advantages, their implementation presents several challenges. The complexity of protocol design, differences in institutional workflows, and the heterogeneity of critically ill patients can all impact the consistency and safety of insulin management by nursing staff. Patients in the ICU frequently experience altered drug metabolism, variable nutritional regimens, such as intermittent enteral feeding or parenteral nutrition, and coexisting conditions like kidney or liver impairment, all of which can significantly disrupt glucose regulation and complicate protocol adherence [23]. Inflexible protocols may not account for such variables, leading to glucose excursions and diminishing their overall effectiveness. Indeed, growing research highlights the critical role of metabolic risk stratification in managing blood glucose levels among critically ill patients. Various clinical factors, such as kidney and liver function, corticosteroid therapy, and methods of nutritional support, have been found to substantially impact glucose stability and fluctuations [24,25,26]. For example, in critically ill patients, glycemic variability has been shown to be independently associated with corticosteroid use and parenteral nutrition, with greater variability linked to a higher risk of mortality [27]. Similarly, studies in mechanically ventilated COVID-19 patients found that those exposed to high cumulative corticosteroid doses (≥320 mg methylprednisolone equivalent) had more frequent episodes of glucose levels falling outside the recommended range, highlighting the destabilizing effect of steroids on glucose control [28]. Additionally, extensive observational research has linked glycemic variability, assessed through measures such as the mean amplitude of glycemic excursion, to increased mortality in the ICU, particularly among patients without a prior diabetes diagnosis [29]. These insights emphasize the need to integrate individualized risk parameters into nurse-driven insulin adjustment protocols. Incorporating such variables into tiered decision-support frameworks could enhance the safety and personalization of glycemic management strategies within critical care settings.

Another limitation lies in the heterogeneity of nursing experience and training. Even within the same institution, the degree of comfort and familiarity with insulin management can vary significantly among staff. Without regular training, validation of competencies, and ongoing quality audits, the potential for protocol misapplication or omission of escalation steps increases [30].

The implementation of nurse-led glycemic control protocols not only reflects a shift in clinical practice but also introduces a complex array of legal, ethical, and professional considerations that must be addressed to ensure safe and effective care. In many healthcare jurisdictions, the authority to initiate or adjust drug therapies, particularly high-risk medications like insulin, remains within the traditional purview of physicians [31]. This legal boundary varies considerably between regions and institutions, directly influencing how, where, and whether such protocols can be adopted.

In some systems, nurse-led insulin titration may be permitted under specific delegation models, while in others, it may be legally restricted, regardless of clinical evidence supporting its safety and efficacy [32,33,34].

While standardized protocols are designed to reduce variability and promote best practices, they cannot account for every clinical nuance encountered at the bedside. Critically ill patients often present with rapidly changing conditions that require nuanced interpretation of protocol parameters, judgment under pressure, and occasionally deviation from strict algorithmic steps, decisions that may place nurses in ethically and legally ambiguous positions, especially when acting autonomously [35]. This creates an inherent tension between maintaining adherence to institutional policy and exercising clinical judgment in real-time, challenging the boundaries of nursing autonomy within medically delegated frameworks. Moreover, the administration of insulin through various routes, subcutaneous, intravenous bolus, or continuous infusion, carries distinct pharmacokinetic profiles, risks, and monitoring requirements [36,37,38,39].

These considerations underscore the need for interdisciplinary collaboration among physicians, nurses, legal experts, hospital administrators, and professional regulatory bodies [40]. A shared understanding of both the clinical rationale and the legal framework surrounding protocol implementation is essential. Embedding this interdisciplinary perspective into protocol development allows healthcare institutions to proactively define delegation models, clarify professional boundaries, and develop educational and risk mitigation strategies. By doing so, they create an operational environment in which nurses can safely exercise clinical judgment within their scope, while ensuring accountability and patient safety. Such policies not only support confident, competent nursing practice but also foster a culture of shared responsibility and improved care quality. In particular, nurses operating under nurse-led glycemic control protocols must have formal authorization from their healthcare institutions, and the protocols themselves require approval from appropriate legal and clinical governance authorities. In regions where the scope of nursing practice is strictly regulated, the lack of explicit legal backing for such protocols can place nurses at risk of liability, even when they adhere to established institutional guidelines [32]. For example, if a nurse following a protocol inadvertently causes severe hypoglycemia leading to neurological damage, questions may arise concerning the adequacy of the protocol’s design, the sufficiency of nurse training, and whether the nurse’s actions exceeded their authorized scope of practice. These legal risks are heightened in situations where documentation is incomplete, physician oversight is not readily available, or the protocols are applied inconsistently across providers or shifts.

## 4. Future Perspectives

Although frequent blood glucose monitoring is commonly thought to increase nursing workload, studies have shown that the use of structured glycemic control protocols does not significantly add to nurses’ workload while maintaining adequate glycemic control [5]. Notably, the integration of continuous glucose monitoring (CGM) systems with digital health platforms for remote management in the ICU has been associated with a reduction in severe hypo- and hyperglycemic events, as well as potential cost savings [41]. Technological advancements have further enabled CGM devices to connect with smartphone applications, allowing real-time glucose tracking, visualization of trend arrows indicating the direction and speed of glucose changes, access to summarized data, and seamless sharing with healthcare professionals [42]. While the cost of CGM devices and their associated supplies remains a significant consideration in hospital adoption, these expenditures may be offset by reductions in hypoglycemia-related complications and hospital length of stay, ultimately lowering healthcare resource utilization. Moreover, CGM may help reduce nursing workload by allowing for continuous glucose surveillance without additional staffing demands. A pragmatic and cost-conscious approach may involve prioritizing CGM use for hospitalized patients who are at highest risk of hypo- or hyperglycemia [41].

While potentially revisiting longstanding debates for legal reasons, recent developments in digital health have underscored the value of integrated systems that merge CGM, Artificial Intelligence (AI) based decision-making tools, and remote clinician input. Although most applications have been explored outside of ICU, the foundational concepts offer promise for adaptation in intensive care settings. As an illustrative case, Lee et al. investigated a digital platform tailored for individuals with type 2 diabetes, which included dietary algorithms, intermittent CGM, and a clinician feedback mechanism. Participants receiving this intervention showed marked improvements in HbA1c levels, along with more pronounced weight reduction when compared to standard care. Despite its outpatient context, this study illustrates how semi-autonomous platforms can effectively guide personalized glucose management while preserving clinician supervision [43]. These principles could be reengineered for ICU settings, using tiered AI-generated insulin dosing proposals that require nurse confirmation, to enhance both safety and adaptability in rapidly changing clinical scenarios. Furthermore, such an approach could empower nursing staff by incorporating predictive modeling, individualized metabolic risk profiling, and mobile technologies that allow for real-time data visualization and family engagement.

## 5. Practical Strategies for the Implementation of Nurse-Led Glycemic Control Protocols

To effectively leverage the advantages of nurse-led glycemic control protocols while minimizing associated risks, a comprehensive, structured, and context-sensitive implementation strategy is essential. Nurse-led insulin management in critical care settings is not merely a clinical innovation, but rather an institutional transformation situated at the intersection of professional role evolution, organizational policy, and medico-legal accountability. This transformation demands alignment between evidence-based practices, regulatory frameworks, and institutional capacity [6]. These protocols represent a form of delegated medical decision-making, in which nurses apply standardized insulin titration algorithms under physician oversight. However, this delegation often occurs in complex regulatory environments where legal boundaries may be ambiguous or inconsistently enforced, potentially exposing both clinicians and institutions to liability.

**Figure 1:**
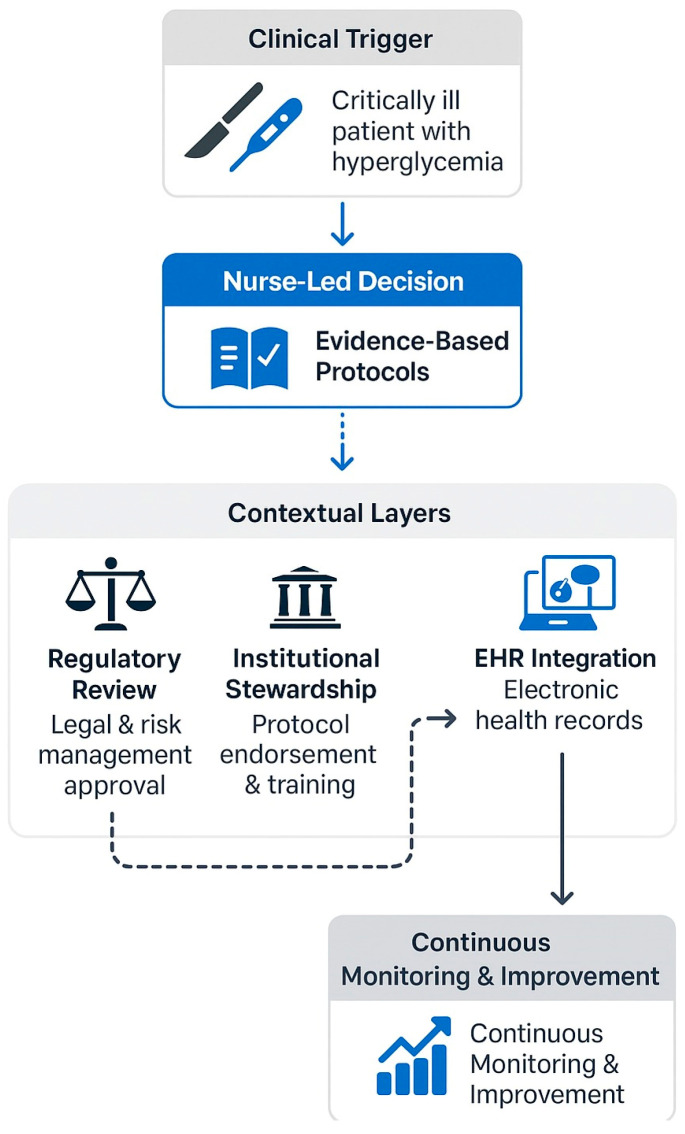
Proposed decision-flow to edit and implement a nurse-led glycemic control protocol.

To address these challenges, we propose a framework based on three interrelated pillars (Figure 1): clinical governance, regulatory clarity, and institutional stewardship. However, before exploring this framework in detail, it is important to emphasize that no single protocol can be universally applied, due to the contextual variability outlined below.

First, clinical governance must ensure that protocols are not only evidence-based but also dynamic, allowing for appropriate flexibility in high-acuity settings where rigid adherence may compromise patient care [44,45]. In particular, regular updates are necessary to incorporate emerging clinical data.

Second, regulatory clarity is fundamental. The wide variability in nurse prescribing laws and protocolized care permissions across jurisdictions can either facilitate or hinder implementation. Institutions must collaborate closely with legal and regulatory bodies to define the limits of nursing autonomy and physician responsibility [32,33,34]. For these reasons, it is important that all protocols must be reviewed and approved by hospital legal and clinical risk management teams to guarantee compliance with relevant nursing practice regulations and insurance policies. Noteworthy, management of complex or atypical glycemic patterns should fall outside the scope of nurse-led protocols, with clear criteria established for escalating care to physicians.

Third, institutional stewardship is critical for long-term sustainability. This includes the establishment of interprofessional implementation teams, comprising nurses, intensivists, endocrinologists, pharmacists, legal advisors, and administrators, to design or adapt insulin protocols tailored to local workflows and patient characteristics [40]. Institutions must also invest in comprehensive education and credentialing programs that cover glycemic physiology, insulin pharmacology, and protocol adherence, supported by simulation-based training and routine competency assessments [46,47,48,49]. Within the training, nurses should also be trained to anticipate hypoglycemic or hyperglycemic episodes [48,49]. To support safe bedside application, decision-support tools such as insulin titration charts, electronic health record integration, real-time alerts, and clear escalation pathways should be implemented to minimize errors and cognitive burden.

Furthermore, robust monitoring systems are essential. Continuous audit-and-feedback loops should be used to track clinical outcomes, protocol adherence, and adverse events, while also creating open feedback channels for frontline staff to report challenges and contribute to iterative protocol refinement. These mechanisms not only ensure compliance but also foster a culture of shared accountability, clinical learning, and institutional transparency.

Finally, it is important to acknowledge that implementation is not a one-size-fits-all process. Local factors such as staffing structures, patient acuity, institutional risk appetite, and technological infrastructure will significantly influence how these protocols are deployed and maintained. By aligning high-level governance with practical, evidence-informed actions, institutions can safely and effectively empower nurses to take a leading role in glycemic management in the ICU, balancing operational efficiency with legal accountability and improving patient care in the process.

## 6. Conclusions

In summary, nurse-led glycemic control protocols present a valuable means to achieve more timely and standardized insulin management in the ICU, potentially improving patient outcomes. When supported by strong clinical governance structures, these protocols can be implemented safely and effectively. Nonetheless, the expansion of nursing roles in insulin titration brings important legal and ethical considerations that must be carefully addressed. Establishing clear institutional policies, maintaining vigilant oversight, and providing continuous education are crucial to ensuring that these protocols fulfill their goal of delivering safe, patient-centered care while protecting all healthcare professionals involved.

## Data Availability

No new data were created or analyzed in this study.
